# Computer literacy among first year medical students in a developing country: A cross sectional study

**DOI:** 10.1186/1756-0500-5-504

**Published:** 2012-09-14

**Authors:** Priyanga Ranasinghe, Sashimali A Wickramasinghe, WA Rasanga Pieris, Indika Karunathilake, Godwin R Constantine

**Affiliations:** 1 Department of Pharmacology, Faculty of Medicine, University of Colombo, Colombo, Sri Lanka; 2 Medical Education and Research Unit, Faculty of Medicine, University of Colombo, Colombo, Sri Lanka; 3 Department of Clinical Medicine, Faculty of Medicine, University of Colombo, Colombo, Sri Lanka

**Keywords:** Computer literacy, Medical undergraduates, Sri Lanka, Developing country

## Abstract

**Background:**

The use of computer assisted learning (CAL) has enhanced undergraduate medical education. CAL improves performance at examinations, develops problem solving skills and increases student satisfaction. The study evaluates computer literacy among first year medical students in Sri Lanka.

**Methods:**

The study was conducted at Faculty of Medicine, University of Colombo, Sri Lanka between August-September 2008. First year medical students (n = 190) were invited for the study. Data on computer literacy and associated factors were collected by an expert-validated pre-tested self-administered questionnaire. Computer literacy was evaluated by testing knowledge on 6 domains; common software packages, operating systems, database management and the usage of internet and E-mail. A linear regression was conducted using total score for computer literacy as the continuous dependant variable and other independent covariates.

**Results:**

Sample size-181 (Response rate-95.3%), 49.7% were Males. Majority of the students (77.3%) owned a computer (Males-74.4%, Females-80.2%). Students have gained their present computer knowledge by; a formal training programme (64.1%), self learning (63.0%) or by peer learning (49.2%). The students used computers for predominately; word processing (95.6%), entertainment (95.0%), web browsing (80.1%) and preparing presentations (76.8%). Majority of the students (75.7%) expressed their willingness for a formal computer training programme at the faculty.

Mean score for the computer literacy questionnaire was 48.4 ± 20.3, with no significant gender difference (Males-47.8 ± 21.1, Females-48.9 ± 19.6). There were 47.9% students that had a score less than 50% for the computer literacy questionnaire. Students from Colombo district, Western Province and Student owning a computer had a significantly higher mean score in comparison to other students (p < 0.001). In the linear regression analysis, formal computer training was the strongest predictor of computer literacy (β = 13.034), followed by using internet facility, being from Western province, using computers for Web browsing and computer programming, computer ownership and doing IT (Information Technology) as a subject in GCE (A/L) examination.

**Conclusion:**

Sri Lankan medical undergraduates had a low-intermediate level of computer literacy. There is a need to improve computer literacy, by increasing computer training in schools, or by introducing computer training in the initial stages of the undergraduate programme. These two options require improvement in infrastructure and other resources.

## Background

Computers are being increasingly utilized as an aid in undergraduate medical education. The use of computer assisted learning has enhanced undergraduate medical education in numerous ways [1]. Computers are used for a wide range of functions in medical education, which ranges from simple drill and practice applications and computer based lectures to more advanced simulations and intelligent tutoring systems [1]. Studies have consistently shown that computer assisted learning improves performance at Multiple Choice Questions (MCQ), Objective Structured Clinical Examination (OSCE) and written assessments [2,3], develops problem solving skills and knowledge [4], and increases student satisfaction [5]. In addition computers are also now being regularly used in student assessments [6]. Furthermore computers are also being increasingly utilized in postgraduate teaching programmes and also plays an essential role in Continuing Medical Education (CME) activities [7]. Hence it is evident that computer literacy has become a vital competency for the present day medical undergraduate.

Computer literacy amongst medical undergraduates in developed countries is at a relatively higher level in comparison to students of developing resource poor countries [8]. Studies have shown that students at resource poor settings lack the necessary skills to use computer-based learning platforms effectively and are therefore at a disadvantage [9]. In addition, a study from Southern India has shown that Indian medical undergraduates use computers frequently for simple tasks, which may not contribute to the development of knowledge acquisition skills [10]. Lack of resources, time and structured training programmes are amongst the reasons for the low computer literacy of medical undergraduates in developing countries [9]. As computers are being widely used, learning to use them to manage knowledge effectively is a core competency in modern medicine. It has been shown that the acquisition of computer skills should commence during the initial stages of the undergraduate curriculum. Studies have shown that medical students who have not acquired basic computer information technology skills by the third year of undergraduate training are unlikely to do so in the final hospital-based years [11].

Sri Lanka is a developing middle-income country in the South Asian region with a population of nearly 20 million [12]. Computers are being used widely by both state and private sector organizations in Sri Lanka including health care services in order to improve productivity. According to recent surveys computer literacy of the Sri Lankan population has increased dramatically over the last few years [13]. Medical education in Sri Lanka is solely conducted at government universities and each year nearly seven hundred students graduate from the seven medical faculties at government universities. Established in 1870 as the Colombo Medical School, the Faculty of Medicine of the University of Colombo, Sri Lanka, is the second oldest medical school in South Asia [14]. The medical undergraduate curriculum at the faculty spans five year, the initial one and a half to two years of pre-clinical training is followed by three years of clinical training [15].

At present there are no studies evaluating computer literacy of Sri Lankan medical undergraduates. At Faculty of Medicine, Colombo computer assisted learning facilities such as Learning Management Systems (LMS) is freely available to students; however, presently there is no structured computer training programme. Due to diverse backgrounds and different levels of exposure, an initiative to introduce Computer Assisted Learning at Faculty of Medicine, Colombo would require a prior assessment of computer-related capabilities and attitudes toward computer-based learning. The present study aims to evaluate the computer literacy among first year medical students at Faculty of Medicine, University of Colombo, Sri Lanka.

## Methods

### Study participants and sampling

The study was conducted at Faculty of Medicine, University of Colombo, Sri Lanka between August and September 2008. One hundred and ninety first year medical students entering the faculty in year 2008 were invited for the study. Informed consent was obtained from each study participant. Ethical approval for the study was obtained from Ethics Review Committee, Faculty of Medicine, University of Colombo, Sri Lanka.

### Study instruments

Data on computer literacy and its potential associated factors were collected by means of an expert-validated pre-tested self-administered questionnaire [see Additional file 1]. The questionnaire evaluated socio-demographic characteristics, computer ownership, computer literacy, attitudes towards computer usage, level of exposure to computers as assessed by prior computer training, preferences for a structured computer training programme at the faculty and tasks for which computers are frequently used.

Computer literacy was evaluated by testing knowledge on 6 different domains; common software packages (MS Word, MS Powerpoint and MS Excel), operating systems (Windows), database management and the usage of internet and E-mail. The questionnaire was designed by two experts in the field of medicine and information technology, and was independently validated by two different experts in the same field. The questionnaire evaluated areas of computer skills that are required by students to acquire new knowledge and perform using computers during the undergraduate curriculum, such as when preparing reports, presentations and conducting research data analysis. The computer literacy section of the questionnaire consisted of 30 different questions. Marks for the individual questions were given taking in to consideration the relative difficulty levels as assessed by two independent experts in the field of computer training and education. The total score for the computer literacy section was sixty-five and the score and number of questions (n) of the different domains were as follows; MS Word – 13 (n = 6), MS Powerpoint – 13 (n = 6), MS Excel – 13 (n = 5), database management – 6 (n = 4), Internet and email – 15 (n = 7) and MS Windows – 5 (n = 2). The final score on computer literacy for each student was calculated as the percentage of the individual score for the six domains out of the total mark of sixty five. Students were categorized in to four categories based on the total marks obtained for the computer literacy questionnaire; low-literacy (<35%), intermediate-literacy (35-49%), high-literacy (50-69%) and very high-literacy (> = 70%).

### Statistical methods

Sri Lanka is a country with nine provinces and twenty five districts. Colombo is the commercial district of Sri Lanka and it is one of the three districts in the Western province. Colombo has the highest computer literacy rate amongst the general population in Sri Lanka [13]. The Faculty of Medicine at the University of Colombo is situated in the ‘Colombo’ district in the ‘Western’ province of Sri Lanka. Thus for analytical purposes the hometown districts and provinces of the students were divided in to two groups ‘Colombo district’ or ‘Other districts’ and ‘Western province’ or ‘Other provinces’. A linear regression was conducted using total score for computer literacy as the continuous dependant variable and the following binary independent variables (unless otherwise stated 0 – No, 1 – Yes); gender (0 – female, 1 – male), province (0 – not Western, 1 – Western), district (0 – not Colombo, 1 – Colombo), Gained knowledge from formal training course, Self learning or Peer learning, doing IT as a subject for GCE (A/L), computer ownership, Usage of computers for word processing, entertainment, presentation, statistics, web browsing, computer programming or computer assisted learning and using internet facility. The ‘explained variance’ of the logistic regression model was calculated by means of Nagelkerke’s R^2^ and the goodness of fit by means of the Hosmer and Lemeshow goodness-of fit test. All data were double entered and cross checked for consistency. Data were analyzed using SPSS version 14 (SPSS Inc., Chicago, IL, USA) statistical software package. A p-value ≤ 0.05 was considered statistically significant.

## Results

One hundred and eighty one students participated in the study (Response rate – 95.3%). Males were 49.7% (n = 90). Forty two percent (n = 76) were from Colombo district and 52.5% (n = 95) were from Western province. Majority of the students (n = 140, 77.3%) owned a computer, there was no significant gender difference observed in computer ownership (Males – 74.4%, Females – 80.2%). The students mostly owned only desktop computers (n = 129, 71.3%), while only a minority (n = 16, 8.8%) of students had both laptop and desktop computers (Table 1). The students have gained their present computer knowledge by either engaging in formal training programme (n = 116, 64.1%), self learning (n = 114, 63.0%) or by peer learning (n = 89, 49.2%). A significant majority of male students have gained their knowledge by peer learning (p < 0.001) (Table 1). However, only 38.1% (n = 69) of the students have taken up IT as a subject at school level. The students that have engaged in formal computer training programmes have mainly completed training programmes on computer applications (n = 108, 59.7%), while only a minority has trained on computer hardware (n = 41, 22.7%), computer programming (n = 40, 22.1%) and web designing (n = 32, 17.7%). There was no significant gender difference in the selection of formal computer training programmes (Table 1).

**Table 1:** Computer ownership, knowledge and exposure to training of students

	**Number (percentage)**			
	**All (n = 181)**	**Males (n = 90)**	**Females (n = 91)**	p*
Ownership of a computer				NS
Desktop computer only	113 (62.4%)	52 (77.6%)	61 (83.6%)	NS
Laptop computer only	11 (6.1%)	7 (10.4%)	4 (5.5%)	NS
Both	16 (8.8%)	8 (11.9%)	8 (11.0%)	NS
Computer knowledge gained by,				
Formal training programme	116 (64.1%)	54 (60.0%)	62 (68.1%)	NS
Self learning	114 (63.0%)	61 (67.8%)	53 (58.2%)	NS
Peer learning	89 (49.2%)	56 (62.2%)	33 (36.3%)	<0.001
Formal training on,				
Computer hardware	41 (22.7%)	22 (24.4%)	19 (20.9%)	NS
Computer programming	40 (22.1%)	20 (22.2%)	20 (22.0%)	NS
Computer applications	108 (59.7%)	51 (56.7%)	57 (62.6%)	NS
Web designing and graphics	32 (17.7%)	16 (17.8%)	16 (17.6%)	NS
IT education at School level,				
Subject in GCE (O/L)	4 (2.2%)	3 (3.3%)	1(1.1%)	NS
Subject in GCE (A/L)	69 (38.1%)	32 (35.6%)	37 (40.7%)	NS

* p values for Males vs. Females, NS – Not Significant, GCE – General Certificate of Education, O/L – Ordinary Level, A/L – Advanced Level.

The students used computers for predominately; word processing (n = 173, 95.6%), entertainment (n = 172, 95.0%), web browsing (n = 145, 80.1%) and preparing presentations (n = 139, 76.8%). Only a few students have used computers more advanced functions such as computer assisted learning (n = 40, 22.1%), computer programming (n = 45, 24.9%) and database management (n = 73, 40.3%). Most of the students were using internet facilities (n = 127, 70.7%), which was mainly accessed from their own residences (n = 83, 45.9%). Only 5 students (2.8%) used internet facilities that were freely available at the faculty. The frequency of internet access varied between students; most accessed 2–3 hours per week (n = 51, 40.2%), followed by rarely (n = 34, 26.8%), 2–3 h per month (n = 25, 19.7%) and 2–3 h per day (n = 17, 13.4%). One hundred and fifteen students had their own e-mail address (63.5%), of which 57.6% (n = 68) checked emails at least once a week.

Majority of the students (n = 137, 75.7%) expressed their willingness for a formal computer training programme at the faculty (Males – 74.4%, Females – 76.9%). The students mostly preferred to be trained by either a computer trainer (n = 68, 49.6%) or peers (n = 40, 29.2%) (Table 2). There was no significant gender difference in this preference. They preferred to be trained on using the internet (n = 114, 83.2%), computer statistics (n = 100, (73.0%), making presentations (n = 73, 53.3%) and word processing (n = 48, 35.0%). A significant majority of females (90.0%) expressed willingness for training in using internet than males (76.1%) (Table 2). Eighty three students (60.6%) wanted the formal training programmed to be scheduled prior to the commencement of faculty teaching activities (Table 2).

**Table 2:** The needs of students that preferred a formal IT training programme at faculty

	**Number (percentage)**			
	**All (n = 137)**	**Males (n = 67)**	**Females (n = 70)**	p*
Prefer learning from,				
Computer trainer	68 (49.6%)	36 (53.7%)	32 (45.7%)	NS
Peers	40 (29.2%)	18 (26.9%)	22 (31.4%)	NS
Senior colleagues	17 (12.4%)	7 (10.4%)	10 (14.3%)	NS
Faculty academic staff	12 (8.8%)	6 (9.0%)	6 (8.6%)	NS
Formal training on,				
Word processing	48 (35.0%)	26 (38.8%)	22 (31.4%)	NS
Presentations	73 (53.3%)	34 (50.7%)	39 (55.7%)	NS
Internet	114 (83.2%)	51 (76.1%)	63 (90.0%)	<0.05
Computer statistics	100 (73.0%)	53 (79.1%)	47 (67.1%)	NS
Timing of training programme,				
Before commencement of faculty teaching	83 (60.6%)	38 (56.7%)	45 (64.3%)	NS
During 1^st^ or 2^nd^ term	25 (18.2%)	13 (19.4%)	12 (17.1%)	NS
Just prior to whenever IT skills are required	29 (21.2%)	16 (23.9%)	13 (18.6%)	NS

* p values for Males vs. Females, NS – Not Significant.

**Figure 1:**
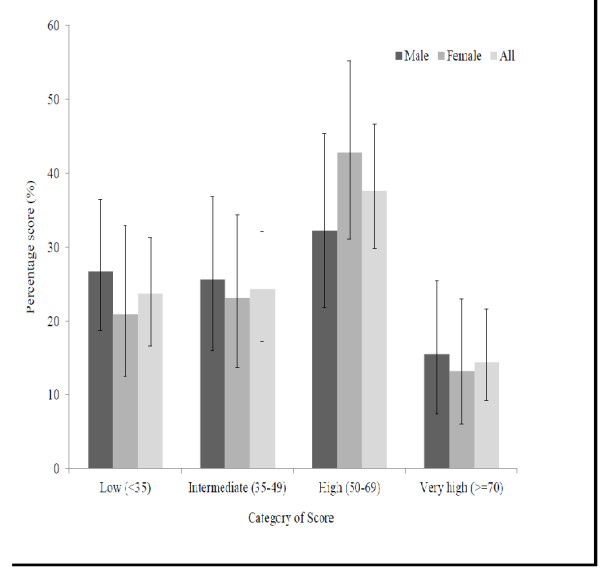
The students’ score range for the computer literacy questionnaire.

The mean score of students out of hundred for the computer literacy section of the questionnaire was 48.4 ± 20.3. There was no significant gender difference in the mean scores (Males – 47.8 ± 21.1, Females – 48.9 ± 19.6). Most student (37.6%) were in the category of high (score 50-69%) computer literacy (Figure 1). However, 47.9% of students had a score less than 50% for the computer literacy questionnaire. There were 14.4% students having a very-high computer literacy (score > =70%). There was no significant gender difference observed in the categories of computer literacy (Figure 1). Students from Colombo district (54.2 ± 19.8) had a significantly higher mean score in comparison to students from other districts (44.2 ± 19.8) (p < 0.001). However, this difference was not observed between males from Colombo district (49.9 ± 22.7) and other districts (46.8 ± 20.4) (p-NS). Students residing in Western province (54.9 ± 18.5) had a higher mean score than those residing in other provinces (41.1 ± 19.8) (p < 0.001). In both genders the difference in mean score between the provinces was significant. Students owning a computer had a higher mean score (50.9 ± 18.9) compared to others (39.5 ± 22.4) (p < 0.001). In addition, students who have gained their present computer knowledge from a formal training course (56.7 ± 16.7) also had a significantly higher mean score than other (33.6 ± 17.7) (p < 0.001). However there was no statistically significant difference observed between score of those who have said that they have gained their knowledge from self-learning or by peer-learning.

The linear regression model explained 55.6% of the variance in score for computer literacy (R^2^ = 0.556). The analysis of variance revealed that the final model was significant (F_17, 2431_ = 12.002, p < 0.001). In all students, undergoing a formal computer training course was the strongest predictor of computer literacy score (β = 13.034, p <0.001), followed by Using internet facility (β = 12.984, p < 0.001), being from Western province (β = 11.744, p < 0.001), using computers for Web browsing (β = 8.041, p < 0.01) and computer programming (β = 8.592, p < 0.001), ownership of a computer (β = 4.609, p < 0.01) and doing IT as a subject in GCE (A/L) examination (β = 4.261, p < 0.05) (Table 3). Being from Colombo district and gender were not significant predictors of computer literacy score (Table 3).

**Table 3:** Results of the linear regression analysis

**Factors**	**ß – coefficient**	**95% Confidence interval**	**p value**
Gender			
Male	0		
Female	−1.581	−6.074 to 2.911	NS
District of residence			
Not Colombo	0		
Colombo	4.095	−0.550 to 9.640	NS
Province of residence			
Not Western	0		
Western	11.744	4.206 to 19.283	<0.001
Gained knowledge from,			
Formal training course*	13.034	7.518 to 18.552	<0.001
Self learning*	1.056	−4.274 to 6.387	NS
Peer learning*	0.439	4.521 to 5.398	NS
IT as a subject for GCE (A/L)*	4.261	0.201 to 8.722	<0.05
Computer ownership*	4.609	2.233 to 6.451	<0.01
Usage of computers for,			
Word processing*	8.155	−3.287 to 19.588	NS
Entertainment*	6.937	−3.064 to 16.940	NS
Presentation*	3.959	−2.549 to 10.468	NS
Statistics*	1.064	−6.459 to 4.329	NS
Web browsing*	8.041	6.773 to 10.857	<0.01
Computer programming*	8.592	2.843 to 14.342	<0.001
Computer Assisted Learning*	3.083	−2.411 to 8.579	NS
Using internet facility*	12.984	6.067 to 19.901	<0.001

* - No = 0, Yes = 1; GCE – General Certificate of Education; A/L – Advanced Level; NS – Not Significant.

## Discussion

The recent technological advances in the fields of medicine and medical education have made computer literacy a vital competency for the present day medical undergraduate. In this first comprehensive survey of computer literacy and its associates amongst Sri Lankan medical undergraduates we demonstrate that the level of computer literacy in Sri Lanka remains at an intermediary stage with nearly half the students obtaining a computer literacy score less than or equal to 50%. In addition, only about 15% of students had very high-computer literacy levels comparable to students of developed countries [8,16]. Hence similar to other developing countries Sri Lankan medical undergraduates could be at a disadvantage in comparison to their counterparts from developed resourceful countries.

A majority of Sri Lankan medical undergraduates (nearly 80%) had their own computers, a figure which is much higher than the recently reported value of 11.4% for the general population of Sri Lanka [13]. This is comparable to computer ownership data of medical undergraduates from developed countries [17]. Computer ownership is an important prerequisite for improving computer literacy; our data demonstrates that computer ownership was a significant predictor of computer literacy amongst the students. The important relationship between computer ownership and computer literacy is also evident by the fact that several medical faculties in developed countries have adopted a mandatory computer ownership criteria for admission [18]. The adoption of a requirement for student ownership of computers at these institutions has successfully facilitated development of students computer skills [18]. Hence, instituting methods such as provision of low-interest loans to increase the ownership of computers amongst Sri Lankan students might help further increase computer literacy.

Formal training of students was also significant predictor of computer literacy. Although our results show that over 60% of students have undergone formal training programmes, only 38% have studies IT as a subject in school. Hence there is a need to increase computer training of students during their school years, in order to bridge the computer skills requirement gap between school and university levels. Another plausible alternative is the implementation of a formal computer training programme during faculty years. Such programmes could also be attractive to students as demonstrated by the high student preference for a formal computer training programme in the present study, this is similar to findings from other resource poor settings [19]. Implementing a formal computer literacy course with stated objectives and measurable outcomes for first-year medical students is one way to instil a minimal level of competence and to target those students who are in need of further instruction [20]. However, such implementation would necessitate the overcoming of barriers such as the finding of adequate time allocations from the busy undergraduate medical curriculum and the lack of adequate resources at medical faculties for computer training. Rapidly evolving use of technology in medical education will require students to acquire a host of new technological skills to meet with future demands, which is in addition to the minimal skills evaluated in the present study. Examples of such skills include the ability to search bibliographic databases to obtain current practice guidelines and research information and participation in web conferences and online teaching programmes. It is the duty of the institution to provide the necessary equipment and facilities required such as webcams, web conferencing tools and software, high speed internet connections and access to the major bibliographic databases. However this could be economically demanding in a developing country such as Sri Lanka.

Our results also show that Sri Lankan medical undergraduates mostly use computers for relatively simple tasks like word processing, web browsing and making presentations. While the general usage of computers would help to increase computer literacy, it is doubtful whether these simple tasks would cater to improving the sometimes complex computer skills that are required for computer assisted learning. Studies have shown that the lack of experience with synchronous and asynchronous online communication, may cause problems when using the collaboration tools included in an LMS [21]. It is important to note that presently the students do not have access to most major bibliographic databases due to the high costs involved. In contrast to some published studies we did not observe a significant difference between males and females in computer literacy and its associated factors [9,16]. This could be due to the high literacy and educational levels of Sri Lankan females. Sri Lanka has the highest female literacy level in the South Asian regions [22].

At present although computer knowledge and competence are not essential requirement for medical undergraduates in Sri Lanka, they are becoming increasingly important due to the reasons such as; Limitation of access to current books and journals and difficulty in obtaining up to date information, which may affect performance at examinations and practice after graduation. Poor computer knowledge can adversely affect the collation and analysis of data as well as the final quality of the research work and after graduation it may have an adverse impact on postgraduate training. The present study contributes to the existing scarce knowledge on computer literacy among medical undergraduates in the South Asian region. This together with other similar regional studies will provide guidance for regional research on interventional training programmes aimed at improving knowledge, based on the identified success predictors.

The present study has several limitations; we did not study the effects of several other factors that could be associated with computer literacy such as the specifics of schooling, language of schooling, and computer literacy and use of computers among parents and siblings, similarly whether it was economic constraint or lack of interest that precluded computer ownership and learning. However, we had to curtail the length of the survey instrument taking in to consideration time constraints of medical students. Hence, a lengthy questionnaire could have compromised the accuracy of essential data. The relatively high response rate of over 95% is a major strength of the present study. In addition, this study is the first comprehensive survey from the Sri Lankan setting. The factors identified during the present study could be used to improve computer literacy amongst medical undergraduates in Sri Lanka and other developing countries with similar student populations. In addition, technology is advancing very rapidly and it is possible that some of these students are using devices and resources that are not being captured in the current survey.

## Conclusions

Sri Lankan medical undergraduates had a low-intermediate level of computer literacy. There is a need to improve computer literacy in students with poor knowledge. This could be done by increasing computer training in the schools, or by introducing a computer training and support programme for these students in the initial stages of the undergraduate programme. These two options require improvement in infrastructure and other resources. As there are students who have very good computer skills an ideal form of teaching/learning model to cater to our requirements will be a peer to peer teaching/learning model.

## Abbreviations

A/L: Advanced Level; CAL: Computer Assisted Learning; CME: Continuing Medical Education; GCE: General Certificate of Education; IT: Information Technology; LMS: Learning Management Systems; MCQ: Multiple Choice Questions; NS: Not Significant; O/L: Ordinary Level; OSCE: Ob jective Structured Clinical Examination.

## Competing interests

The authors declare that they have no competing interests.

## Authors’ contributions

PR, GRC, IK and SAW made substantial contribution to conception and study design. SAW, PR and WARP were involved in data collection. WARP, GRC, IK were involved in refining the study design, statistical analysis and drafting the manuscript. PR and GRC critically revised the manuscript. All authors read and approved the final manuscript.

## Authors' information

Ranasinghe P: Is a MBBS graduate at the Faculty of Medicine, University of Colombo. He is a Lecturer at the Department of Pharmacology, Faculty of Medicine, University of Colombo, Sri Lanka. His research interests include Medical Education and Non-communicable diseases.

Wickramasinghe SA: Is a MBBS graduate at the Faculty of Medicine, University of Colombo. She has served as a Research Assistant at the Medical Education Development and Research Centre (MEDARC), Faculty of Medicine, Colombo, Sri Lanka.

Pieris WAR: Is a MBBS graduate at the Faculty of Medicine, University of Colombo. She has served as a Research Assistant at the Medical Education Development and Research Centre (MEDARC), Faculty of Medicine, Colombo, Sri Lanka. Her research interests include Medical Education.

Karunathilaka I: Is the Director of the Medical Education Development and Research Centre (MEDARC), Faculty of Medicine, Colombo, Sri Lanka. He received training in Medical Education at the Centre for Medical Education, University of Dundee. He has authored many publications in medical education and has been a resource person at national and international forums.

Constantine GR: Is a Senior Lecturer at the Department of Clinical Medicine, Faculty of Medicine, University of Colombo. He is a consultant cardiologist and his research interest includes Medical Education and Non-communicable diseases.

## Supplementary Material

Additional file 1 Study questionnaire. Click here for file
