# Supplementary material for: Computer literacy among first year medical students in a developing country: A cross sectional study
Source: BMC Res Notes. 2012 Sep 14;5:504. doi: 10.1186/1756-0500-5-504 (PMC3517310; doi:10.1186/1756-0500-5-504)
Supplement: Additional file 1 — Study questionnaire. [file 1756-0500-5-504-S1.pdf]

Medical Students' Basic Computer Knowledge Assessment

Faculty of Medicine, Colombo

Answer all the questions
Circle the correct response in the "yes / no" questions and tick the best response in the questions with empty cages.

**Gender**	**Male**	**Female**

Home town ____________________

The city where you studied for the A/Ls ____________________

1. Attitude and practice regarding Information Technology

1.1 From where did you gain your current knowledge regarding computers?

A formal training course	Yes	No
An informal independent study	Yes	No
Friends and colleagues	Yes	No

1.2 Have you had a course in the following computer related areas before coming to Medical Faculty?

Computer Hardware	Yes	No
Computer Software	Yes	No
Computer Programming, Systems Analysis & Design	Yes	No
Computer Graphics and Web Designing	Yes	No

Other (specify) ____________________

1.3 Have you done IT as a subject in O/Ls?	Yes	No

If yes, grade obtained ____________________

1.4 Have you done IT as a subject in A/Ls? (General Information Technology)	Yes	No

If yes, grades obtained ____________________

1.5 Which of the following activities have you carried out using a computer?

Preparing a document/ letter	Yes	No
Playing a computer game	Yes	No
Preparing a presentation	Yes	No
Organizing information using a computerized database (For example using Access, Oracle, SQL, etc…)	Yes	No
Using the World Wide Web to search for information	Yes	No
Using a computerized spreadsheet (E.g.: MS Excel)	Yes	No
Writing a computer programme (For example using Visual Basic, Java, C, PERL, etc…)	Yes	No
Using a Computer Assisted Instructions program	Yes	No

Other (specify) ____________________

1.6 Would you like to have an IT educational programme during the medical course?

Yes	No

1.7 If so whom would you mostly prefer to conduct the teaching activities?

- Seniors ☐
- Peers (colleagues) ☐
- Academic staff ☐
- General IT trainers ☐

1.8 If yes to 1.6 when would you think is the best time to conduct such an activity?

- During the English course ☐
- During 1-2 terms ☐
- Just before some activity where IT knowledge is needed (POWER POINT before the presentations or WORD before preparing a project report) ☐
- Other (specify) ____________________

1.10 If yes to 1.6 which programmes would you prefer to learn about?

Microsoft Word	Yes	No
Microsoft PowerPoint	Yes	No
Searching & using Medical Resources on the Internet	Yes	No
Statistical analysis software	Yes	No

Other (specify) ____________________

1.11 Do you own a computer? | Yes | No |

If yes,

What type (Circle one) | Desktop | Lap | Both |

What brand/s and model/s? ____________________

1.12 Do you have an e-mail address? | Yes | No |

If yes, how frequently do you check it/ send mails?

- Daily ☐
- Several times a week ☐
- Once a week ☐
- Once a month ☐
- Rarely ☐

1.13 Do you use internet facilities? | Yes | No |

If yes, from where do you gain access to internet?

Your home	Yes	No
At a friends'/ relatives' home	Yes	No
Internet café	Yes	No
Faculty	Yes	No

How frequently do you use it?

- 2-3 hours a day ☐
- 2-3 hours a week ☐
- 2-3 hours a month ☐
- Rarely ☐

2. **Knowledge on word processing**

2.1 Have you used Microsoft Word before?

Yes	No

2.2 If so, for which activity did you use it?

____

2.3 Microsoft Word offers pre-developed designs for different types of documents such as reports and letters. What are these pre-developed designs called. (circle the correct answer)

Document templates	Web pages	Layout wizards	Tables

2.4 Which of these icons would you use to delete text from a page and store it on the Clipboard (Circle the correct icon)

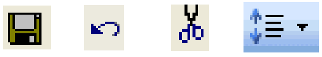

2.5 You have prepared a document using Microsoft word. Next you want to get all the lines to start from the left side of the page. How would you do it?

Select the document and click on the 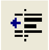 icon

Select the document and click on the 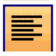 icon

Go to **Format,** then select **Font** and select **left alignment**

Select the document and click on 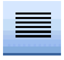 icon

2.6 What do these icons mean

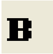 ______________________________

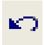 ______________________________

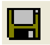 ______________________________

3. **Knowledge on Power Point**

3.1 Have you used Microsoft Power Point before?

Yes	No

3.2 If so, for which activity did you use it?

____

3.3 What is the keyboard shortcut you can use to start a presentation/ slide show (Circle the correct answer)

F1	F5	F8	Shift

3.4 What do these icons mean

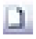 ______________________________

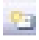 ______________________________

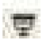 ______________________________

3.5 Which of the following can be used to insert a new slide?

Right-click on the slide that you want the new slide to follow, and then press ENTER.

By clicking on the 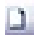 icon.

Select the entire presentation and press ENTER.

On the **Slides** tab in the left of the window, Right-click the slide thumbnail that you want the new slide to follow, and then click **New Slide** on the shortcut menu.

3.6 Microsoft PowerPoint offers pre-developed designs for different types of presentations. What are these pre-developed designs called? (circle the correct answer)

**Design Templates**	**Slide Image**	**Design Insert**	**Slide Sorter**

4. Knowledge on Microsoft Office Excel

4.1 Have you used Microsoft Excel before?

**Yes**	**No**

4.2 If so, for which activity did you use it?

____

4.3 The address of the cell in Microsoft Excel (please tick the best answer)

Consists of row heading followed by the column heading

Is the column heading

Consists of column heading followed by the row heading

Is the name you give for each cell

4.4 What do these icons mean

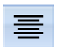 ____

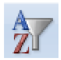 ____

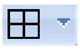 ____

4.5 You have entered a set of values in a column. Now you want to get the sum at the bottom of the column. How would you do it?

Select all the values and click on the + key on the keyboard.

Select all the values and click on **Auto sum** icon.

Select one value, click on **Auto sum** icon and repeat it for all the values.

Go to **Tools**, select the **Calculator** option and do the calculations

5 Knowledge on Data management

5.1 Following software can be used for Data analysis

- Macromedia Flash
- SPSS
- Adobe Acrobat Reader
- Mind Manager

5.2 Which variable type is best used to store the following data (circle the correct answer)

The height of a person	Date	String	Numeric
A date	Date	String	Numeric
An address	Date	String	Numeric

6. Using the Internet and e-mail

6.1 Which of the following software products might you use to view web pages?

- Outlook Express
- Mozilla Firefox
- Adobe Acrobat Reader
- Safari

6.2 You have gone to the faculty web site and clicked on "About faculty" link on the home page. You now want to return to the home page. Which button would you click?

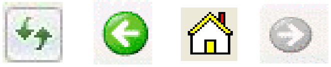

6.3 If you did not know the URL for Faculty of Medicine, you could use a search service to locate the Faculty of Medicine, Colombo web site. Which of the following would you enter in the search box to do this?

- **Faculty of Medicine and Colombo**
- **Faculty of Medicine Colombo**
- **"Faculty of Medicine Colombo"**
- **Faculty and Medicine and Colombo**

6.4 You wish to access information regarding "Anatomy – 1st Week of development and implantation" lecture. What key words should you use for best access?

- Development, implantation
- 1st week of development
- Anatomy, 1st week, development
- Embryology, 1st week of development

6.5 Which of the following is an example of an e-mail address?

- www.yahoo.com
- clerkc@mfc.edu
- Arthur C. Clerk
- Arthurclerk

6.6 What are the web sites you could use for accessing scientific information

- Pub med
- Elakiri.com
- Scientific American
- BBC world

6.7 A word processing file can be attached to an e-mail message

**Yes**	**No**

7. Knowledge on Operating Systems

7.1 Creating a subdirectory means that you have to

- Create a new folder for the specific files
- Place the specific files on your Desktop
- Save all files on your hard drive
- Place the specific files on My Documents

7.2 You have been preparing an important document. You close the document by mistake and want to reopen it. You cannot remember where you have saved it or the exact file name. How can you retrieve it most easily?

- Click on **Start**, then go to **Search.**
- Click on **Start**, then go to **My Recent Documents**.
- Click on **Start**, then go to **Control Panel.**
- Click on **Start**, then go to **Help and Support**.
